# A Metabolomics Approach to Stratify Patients Diagnosed with Diabetes Mellitus into Excess or Deficiency Syndromes

**DOI:** 10.1155/2015/350703

**Published:** 2015-01-18

**Authors:** Tao Wu, Ming Yang, Tao Liu, Lili Yang, Guang Ji

**Affiliations:** ^1^Center of Chinese Medicine Therapy and Systems Biology, Shanghai University of Traditional Chinese Medicine, Shanghai 201203, China; ^2^Institute of Digestive Disease, Longhua Hospital, Shanghai University of Traditional Chinese Medicine, Shanghai 200032, China

## Abstract

The prevalence of type 2 diabetes continuously increases globally. The traditional Chinese medicine (TCM) can stratify the diabetic patients based on their different TCM syndromes and, thus, allow a personalized treatment. Metabolomics is able to provide metabolite biomarkers for disease subtypes. In this study, we applied a metabolomics approach using an ultraperformance liquid chromatography (UPLC) coupled with quadruple-time-of-flight (QTOF) mass spectrometry system to characterize the metabolic alterations of different TCM syndromes including excess and deficiency in patients diagnosed with diabetes mellitus (DM). We obtained a snapshot of the distinct metabolic changes of DM patients with different TCM syndromes. DM patients with excess syndrome have higher serum 2-indolecarboxylic acid, hypotaurine, pipecolic acid, and progesterone in comparison to those patients with deficiency syndrome. The excess patients have more oxidative stress as demonstrated by unique metabolite signatures than the deficiency subjects. The results provide an improved understanding of the systemic alteration of metabolites in different syndromes of DM. The identified serum metabolites may be of clinical relevance for subtyping of diabetic patients, leading to a personalized DM treatment.

## 1. Introduction

Diabetes mellitus (DM) is a chronic disease defined with high blood glucose levels, which may be due either to the progressive failure of pancreatic *β*-cell function and consequently a lack of insulin production (type 1: T1DM), or to the development of insulin resistance and subsequently the loss of *β*-cell function (type 2: T2DM). DM affects more than 230 million people worldwide and T2DM is predicted to affect approximately 8% of the population by 2030 [1]. The chronic hyperglycemia of diabetes is associated with significant long-term sequelae, particularly damage and/or dysfunction and failure of various organs, especially the kidneys, eyes, nerves, heart, and blood vessels [2]. Both the macrovascular (coronary artery disease, peripheral artery disease, and stroke) and microvascular (retinopathy, nephropathy, and neuropathy) complications are the major causes of morbidity and mortality of diabetes.

Traditional Chinese medicine (TCM) has a long history and particular advantages in the diagnosis and treatment of diabetes mellitus. Syndrome differentiation is not only the basic unit of TCM theory, but also the bridge to associating disease and formula. TCM can stratify the diabetic patients based on their different TCM syndromes and, thus, allow a personalized treatment. When people suffer from a disease, Yin (things associated with the physical form of an object), Yang (things associated with energetic qualities), Qi (life force that animates the forms of the world), and Xue (dense form of body fluids that have been acted upon and energized by Qi) [3] are in an abnormal state. Similarly, DM could be classified as having deficiency syndrome or excess syndrome, which refers to the insufficiency or excess in Qi, Xue, Yin, and Yang. However, syndromes depend on medical experience, academic origins, and other factors so that the concept of syndromes is vague and broad, which makes clinical application difficult. Hence, it is more important to realize the syndrome objectification and standardization. Furthermore nowadays although the diagnosis and treatment of manifest diabetes have been thoroughly investigated, the identification of novel pathways or biomarkers indicative of the TCM syndrome differentiation of diabetes is still underway.

With the rapid development of the analytical technology and advanced multivariate statistical and bioinformatic tools, metabolomics has become a promising approach for understanding and elucidating the etiology and mechanisms of human diseases [4–7] and has been extensively applied to life science [8–10]. Metabolomics is also able to provide metabolite biomarkers for disease subtypes. The growing research field of metabolomics has introduced new insights into the pathology of diabetes as well as methods to predict disease onset and has revealed new biomarkers during the last decade. Recent epidemiological studies first used metabolism to predict incident diabetes and revealed branched-chain and aromatic amino acids including isoleucine, leucine, valine, tyrosine, and phenylalanine as highly significant predictors of future diabetes [11, 12]. Our previous work also showed urinary carbohydrate metabolic characterization of DM patients with different traditional Chinese medicine syndromes, including biomarkers different from non-DM patients [13]. Xu et al. found that three TCM syndromes including Qi-deficiency, Qi and Yin-deficiency, and damp heat can be separated using metabolomics technology and such differences can be manifested by plasma fatty acids and lipid parameters [14]. Wei et al. designed an explorative study of 50 prediabetic males, and finally they indicated more disturbances of carbohydrate metabolism and renal function in subtype “Qi-Yin deficiency with stagnation” compared with “Qi-Yin deficiency with dampness” [15]. However, it is still far from clear about the different syndrome of diabetes although so many investigations have been performed [16].

In this study, we applied a metabolomics approach using ultraperformance liquid chromatography (UPLC) coupled with quadruple-time-of-flight (QTOF) mass spectrometry to characterize the metabolic alterations of different TCM syndromes including excess and deficiency in patients diagnosed with DM and discover biomarkers using metabolomics technology to further find the deep connotation of TCM syndromes.

## 2. Methods

### 2.1. Study Population

DM patients were prospectively included from the Tianlin Community health center, Shanghai city of China, during August 2009 to May 2010. DM is characterized by a fasting plasma glucose (FPG) of ≥7.0 mmol/L, a post-load plasma glucose (2 h PG) of ≥11.1 mmol/L, or a history of oral hypoglycemic or insulin use, or both, based on the standard formulated by the World Health Organization in 1999 [17]. TCM syndromes, including deficiency and excess syndromes, were differentiated according to the guidelines [18]. Patients suffering from other serious diseases involving major organs or infective diseases were excluded from the study. Patients with deficiency and excess syndrome simultaneously were also excluded. The detailed inclusion and exclusion criteria were shown in previous work [13]. Altogether 295 subjects with T2DM (238 deficiency and 57 excess samples) were recruited to the study. All subjects provided their written informed consent. The ethics committee of the hospital approved the study plan and the study complied with the Declaration of Helsinki.

### 2.2. Biochemical Analysis

Body mass index (BMI) was calculated as weight (kg) divided by height (m) squared. The waist-hip ratio (WHR) was defined as the waist circumference (cm) divided by the hip circumference (cm). Systolic blood pressure (SBP) and diastolic blood pressure (DBP) were measured using standard mercury sphygmomanometers on the right arm of seated participants. Serum fasting glucose, triglyceride (TG), high density lipoprotein (HDL-C), very low-density lipoprotein cholesterol (VLDL-C), alanine aminotransferase (ALT), and 2 h PG were analyzed using an automatic bioanalyzer (Hitachi7180, Tokyo, Japan). Liver ultrasound examination was carried out on the same equipment (Aloka1700, Japan).

### 2.3. Chemicals

HPLC grade methanol, acetonitrile, and formic acid were purchased from Merck Chemicals (Darmstadt, Germany). L-chlorophenylalanine was purchased from Sigma-Aldrich (St. Louis, MO). Ultrapure water was produced by a Milli-Q water system (Millipore, Billerica, USA).

### 2.4. Serum Sample Preparation for UPLC-QTOFMS

Fasting serum samples were obtained and prepared strictly according to the previous work [19]. An aliquot of 100 *μ*L of serum was mixed with 400 *μ*L of a mixture of methanol and acetonitrile [5 : 3, (containing 0.1 mg/mL L-chlorophenylalanine as the internal standard)]. The mixture was then vortexed for 2 min, allowed to stand for 10 min, and centrifuged at 14 500 g for 20 min. The supernatant was used for UPLC-QTOFMS analysis.

### 2.5. UPLC-QTOFMS Spectral Acquisition of Serum Samples and Data Preprocessing

A Waters ACQUITY ultraperformance liquid chromatography (UPLC) system equipped with a binary solvent manager and a sample manager (Waters Corporation, Milford, MA, USA), coupled to a QTOF mass spectrometry with an electrospray interface (Waters Corporation, Milford, MA), was used throughout the study as aforementioned [19]. All Chromatographic separations were performed with an ACQUITY BEH C18 column (1.7 *μ*m, 100 × 2.1 mm internal dimensions, Waters). The column was maintained at 50°C, and the injection volume of all samples was 5 *μ*L. The LC elution conditions were optimized as follows: linear gradient from 1 to 20% B (0-1 min), 20 to 70% B (1–3 min), 70 to 85% B (3–8 min), 85 to 100% B (8-9 min), and isocratic at 100% B (9–9.5 min) with a flow rate of 0.4 mL/min. (A) Water with 0.1% formic acid and (B) acetonitrile with 0.1% formic acid were used for positive ion mode (ESI+), while (A) water and (B) acetonitrile for negative ion mode (ESI−). The mass spectrometer was operated with source and desolvation temperatures set at 120°C and 300°C, respectively. The desolvation gas was set at a flow rate of 600 L/hr. The capillary voltage was set of 3.2 and 3 kV and the cone voltage of 35 and 50 V, respectively, in the positive and negative ion modes.

The UPLC-MS raw data were processed using MarkerLynx 4.1 (Waters, Manchester, UK) using parameters mentioned in the previous work [20–23]. After removing the ion peaks generated by the internal standard, the data were normalized by dividing the sum of all peak intensities within the sample and then a data matrix consisted of the retention time, *m*/*z* value, and the normalized peak area was exported for multivariate statistical analysis using the K-OPLS package (available at http://kopls.sourceforge.net/download.shtml) and Statistics toolbox of the Matlab (version 7.1, Mathwork Inc.) software. Compound annotation was carried out by comparing the retention time, molecular weight, preferred adducts, and in-source fragments based on our in-house reference standard library (−800 mammalian metabolite standards available) and web-based resources, including the Human Metabolome Database (http://www.hmdb.ca/).

### 2.6. Data Analysis

Data from the common and clinical information were expressed as mean ± standard deviation (S.D.). Differences between the means of groups were analyzed using independent samples *t*-test for continuous variables and Pearson chi-square tests for categorical variable using the SPSS 17.0 software (SPSS, Chicago, Illinois, USA), with a two-sided *P* value of <0.05 considered statistically significant.

By applying preprocessing methods, both a synthetic minority oversampling technique (SMOTE) bagging rebalancing method and a genetic algorithm (GA) with kernel-based orthogonal projections to latent structures (K-OPLS), differential metabolites between groups from the UPLC-QTOFMS data were observed. The major protocol was according to our previous work [13, 24] and related literatures [25, 26], as shown in Figure S1 (see Supplementary Material available online at http://dx.doi.org/10.1155/2015/350703).

First, for the reason of the data's unbalance, the SMOTE algorithm was performed to simulate small data and achieve the equilibrium of the whole data set. According to the class of 50% randomly selected samples as training set, the original sample data as test set, and GA-KOPLS algorithm with balanced prediction errors of test set as a fitness function, the nearest neighbor parameters “*n*” of SMOTE were optimized.

Secondly, under the optimized SMOTE parameter, GA-KOPLS algorithm is applied to modeling and selects the important variables (metabolites) at the same time with the balanced prediction error in test set as the fitness function (minimize error). Evaluation of classifier accuracy during each GA run was performed using a cross-validation [27].

Thirdly, the important variables selected by the GA were applied to a K-OPLS algorithm for classification, and the parameters including the Gaussian kernel function parameter (*σ*) and the number of Y-orthogonal components (Ao) of the K-OPLS model were optimized with internal tenfold cross-validation of training set. The kernel matrix *K* was centered to model estimation. The samples from each training set study were taken for classification, in turn, excluding those being classified from the selected samples in the training set. The prediction accuracy of the original data set, AUC, sensitivity, and specificity were used to evaluate the K-OPLS model performance. Details on the model were provided in the previous work [13].

Finally, the frequency of variable significance test was performed by GA, and the *P* values were calculated based on the binomial probabilities of variables being selected in the 50 independent runs, to identify the metabolites with significant influence in the classification. One has

(1) $$\begin{array}{c} P\left(x\right)=\overset{n}{\underset{i=x}{\sum }}\left(\begin{array}{c} n\\ i \end{array}\right){\left(\frac{m}{v}\right)}^{i}{\left(1-\frac{m}{v}\right)}^{n-i}, \end{array}$$

where *n* = number of runs, *v* = number of variables, and *m* = mean number of times variables are selected, rounded to an integer. For details, see literature [28]. In addition, these metabolites selected from the model were validated at a univariate level with nonparametric Wilcoxon rank sum test with a critical *P* value usually set to 0.05.

## 3. Results

### 3.1. Clinical Characteristics of Patients

Subjects' clinical characteristics of the three groups were summarized in Table 1. The clinical characteristics of this subset of subjects did not differ significantly between the groups at baseline, except for age, BMI, and the coincidence of fatty liver disease, which were significantly higher in the deficiency group than in the excess group.

### 3.2. UPLC-QTOFMS Analysis of Serum Metabolite Profiles

The ESI positive ion mode was more efficient with a significantly greater number of serum metabolites detected than the ESI negative ion mode and, therefore, was selected for the full scan detection mode. Among a total of approximately 6680 metabolite features obtained from the UPLC-QTOFMS, 133 metabolites were identified with our in-house reference standard library and further verified by available reference standards. Their peak areas were integrated for further multivariate analysis.

### 3.3. Classification of the K-OPLS Models

**Figure 1:**
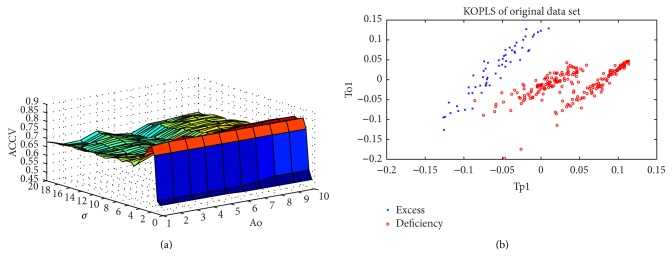
Accuracy of classification of cross-validation (ACCV) (a) and first predictive and *Y*-orthogonal score components (b) by the K-OPLS model in DM patients with excess and deficiency syndrome.

In the present study the nearest neighbor parameter “*n*” of SMOTE was 3 after optimization, from DM patients with excess or deficiency syndrome. A K-OPLS model was fitted using the Gaussian kernel function with the important variables selected from GA. The parameters of GA including initiate population, *K* (times of genetic algebra), selective ratio of initiate variable, and probability of simple point crossover were 30, 150, 0.1, and 0.7, respectively (Table 2). Accuracy of classification of cross-validation (ACCV) was calculated for each combination of *σ* and Ao which were optimized using 10-fold cross-validation. ACCV was the largest when *σ* = 2.5 and Ao = 3 for DM patients with excess and deficiency syndrome (Figure 1(a)).

Table 2 showed the R2X, R2Y, Q2Y, AUC, sensitivity, and specificity used in evaluating all the calibration models of the two groups. R2Xcum and R2Ycum represented the cumulative sum of squares of all the X's (metabolic data) and Y's (disease category data) explained by all extracted components. Q2Ycum is an estimate of how well the model predicts the Y's [28]. High coefficient values of R2Y and Q2Y represent good prediction [29]. As displayed by the score plots of K-OPLS (Figure 1(b)), the two sample groups can be separated into distinct clusters to indicate the changes in the metabolic response of serum samples from the DM patients with excess and deficiency syndrome.

The model statistics R2X = 0.425, R2Y = 1.000, and Q2Y = 0.944 in the model suggest a highly predictive and general model (Table 3). Because the nonlinear method was used in the present study, the R2X had less significant meanings. On the contrary, the major indicator is AUC to evaluate the models' accuracy in nonlinear method. AUC = 0.968 (95% confidence interval = 0.950–0.987) predicted that the models had high accuracy (Table 3).

### 3.4. Representative Differential Metabolites Based on Multivariate and Univariate Analysis

**Figure 2:**
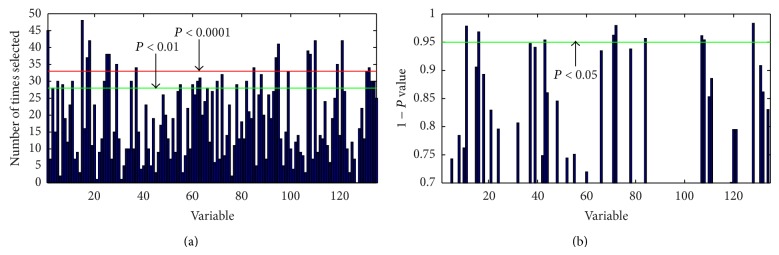
GA runs (a) and Wilcoxon rank sum test (b) in DM patients with excess and deficiency syndrome. (a) *X*-axis presents 135 metabolites as variables; *Y*-axis presents the number of selected times of the variables from GA. (b) *X*-axis presents 135 metabolites as variables; *Y*-axis presents the value of 1 − *P* from Wilcoxon rank sum test.

**Figure 3:**
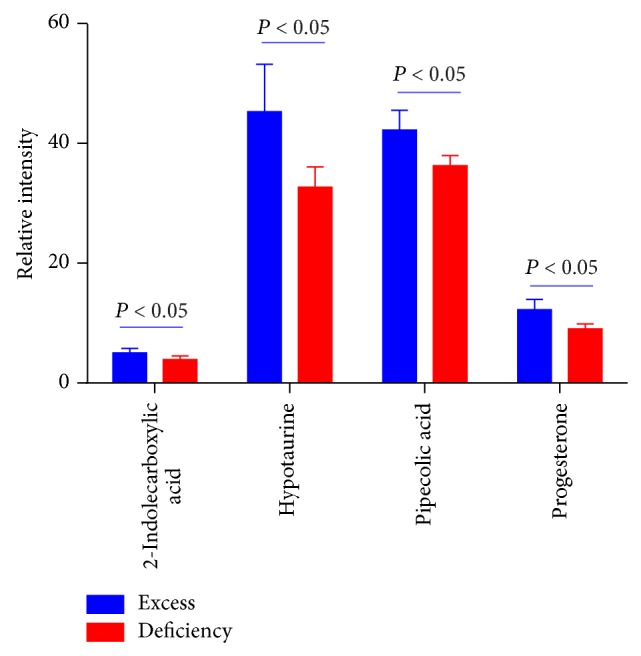
Differentially expressed metabolites between groups in DM patients with excess and deficiency syndrome combined with GA runs and Wilcoxon rank sum test.

The metabolites contributed for the separation between groups derived from UPLC-QTOFMS analysis were selected in accordance with the criteria of multivariate statistics (GA, *P* < 0.001, Figure 2(a)) and nonparametric univariate statistics (Wilcoxon rank sum test, *P* < 0.05, Figure 2(b)). Four differentially expressed metabolites including 2-indolecarboxylic acid, hypotaurine (HTAU), pipecolic acid, and progesterone between DM patients with excess and deficiency syndrome were found (Figure 3). The serum levels of those four metabolites were higher in DM patients with excess syndrome than those with deficiency syndrome.

## 4. Discussion

Serum patterns of metabolites reflect the homeostasis of the organism to some extent. Metabolomics, a discipline dedicated to the global study of metabolites, may deepen our understanding of human health and diseases. In the present study, we found that four metabolites can differentiate two different TCM syndromes in DM, which cannot be characterized by the clinical biochemical indicators. The clear separation between two groups by TCM symptoms and metabolic profiles illustrated that excess and deficiency syndrome had their own substance fundaments.

Clinical characteristics of this subset of subjects in Table 1 showed there was no significant difference between the deficiency and excess groups at baseline, in terms of sex, WC, HC, WHR, DBP, TG, ALT, VLDL, and HDL levels, except for age, BMI, and the coincidence of fatty liver disease, which were significantly higher in the deficiency group than in the excess group. The higher age in deficiency group is in accordance with clinical TCM theory that Qi, Xue, Yin, and Yang are more insufficient in older than in younger persons. This result shows that the clinical biochemical indicators are difficult to differentiate the TCM syndromes. Hence novel approaches for differentiating syndromes are urgently needed. The nontarget metabolomics provides a global view of the organism and also provides an opportunity to stratify the different TCM syndromes like we performed before [13].

In the present study, we performed UPLC-QTOFMS-based serum metabolic profiling combined with GA-KOPLS analysis on DM patients with different syndromes and four metabolites were eventually found between the two TCM syndromes. In order to exclude the effect of age and BMI, we separated two subsets with the cutoff of 70 in age, 25 in BMI. We found that there was no significant difference of the four differential metabolites between groups with Mann-Whitney Test Analysis (*P* > 0.05, Supplementary Table S1). Furthermore, we performed the KOPLS model based on the same parameters. It was shown that no matter the age >70 or ≤70 (*n* = 161 versus 134), deficiency group and excess group could be distinctly separated on the classification (Supplementary Figure S2). Similar results were also found in BMI ≥25 or <25 (*n* = 153 versus 142) (Supplementary Figure S3). Those results prompt that the age and BMI with significant difference between deficiency and excess groups do not affect our final metabolomics results.

2-Indolecarboxylic acid similar to melatonin is a strong inhibitor of lipid peroxidation. Štětinová et al. performed an in vitro study with a standard lipid peroxidation assay, and they finally found that tested drugs inhibited lipid peroxidation in the order of tryptamine (59%) > 2-indolecarboxylic acid (38%) > indomethacin (26%) > melatonin and indole-3-carboxylic acid (13%) [30].

HTAU is a product of enzyme cysteamine dioxygenase in taurine and hypotaurine metabolic pathway. It may function as an antioxidant and a protective agent under physiological conditions [31, 32], and it results in the prevention of peroxynitrite-induced tyrosine nitration to 3-nitrotyrosine and oxidation to dityrosine. Nitration and oxidation of tyrosine residues in proteins have been detected in several conditions of oxidative stress that involve the overproduction of NO^+^ and oxygen radicals. Hence, it is tempting to postulate that the protection afforded by HTAU on tyrosine modification may have important physiological significance. Gossai and Lau-Cam compared taurine, aminomethanesulfonic acid, homotaurine, and HTAU for the ability to modify indices of oxidative stress and membrane damage associated with T2DM. Relative to control values, taurine and its congeners had equiproctective roles in reducing membrane damage, the formation of intracellular malondialdehyde and oxidized glutathione, and the decreases in reduced glutathione and antioxidative enzyme activities in diabetic erythrocytes [33].

Pipecolic acid (piperidine-2-carboxylic acid), the carboxylic acid of piperidine, is a small organic molecule which accumulates in pipecolic acidemia. It is a metabolite of lysine found in human physiological fluids such as urine and serum. However, it is uncertain whether pipecolic acid originates directly from food intake or from mammalian or intestinal bacterial enzyme metabolism.

Progesterone, converted from pregnenolone, serves as an intermediate in the biosynthesis of gonadal steroid hormones and adrenal corticosteroids. Progesterone was observed to have antioxidant properties, reducing the concentration of oxygen free radicals [34]. Recently, a crosstalk between progesterone and melatonin has been observed in various preclinical studies. The melatonin is reported to increase progesterone level and expression of progesterone receptors in reproductive tissues [35].

Interestingly, the above four metabolites are all related to oxidative stress. We suggest that diabetic patients with excess syndrome may have more severe systemic oxidative stress than those with deficiency syndrome. Understanding syndromes is a core research to develop more efficient therapeutic strategies, classification, and diagnostic criteria for patients. It will contribute to TCM syndrome objectification and standardization to the better diagnosis and therapy of disease. Further investigations with larger sample sizes are needed to confirm our findings.

## 5. Conclusion

The present study provides an improved understanding of the systemic alteration of metabolites in different syndromes of DM. It also presents that metabolomics method would be helpful in establishing a suitable model for reasonably evaluating disease syndrome, exploring pathological mechanism of syndrome, and clarifying the relationships between the syndrome and related diseases. Furthermore, the identified serum metabolites may be of clinical relevance for subtyping of diabetic patients, leading to a personalized DM treatment.

## Supplementary Material

Figure S1 presents an overview of data analysis in the manuscript. Table S1 shows there is no significant difference of the four differential metabolites between groups with Mann-Whitney Test Analysis (P > 0.05). Furthermore, the KOPLS model also shows that no matter the age > 70 or ≤ 70 (n = 161 versus 134), deficiency group and excess group could be distinctly separated on the classification in Figure S2. Similar results are also found in BMI ≥ 25 or < 25 (*n* = 153 versus 142) in Figure S3.). The results prompts that the age and BMI with significant difference between deficiency and excess groups do not affect the final metabolomics results.

## Figures and Tables

**Table 1:** Clinical characteristics of excess and deficiency syndromes in patients with DM (mean ± SD).

	Patients with DM (*n* = 295)			
	Total	Excess	Deficiency	*P* ^a^
Gender (*n*, male/female)	295 (107/188)	57 (23/34)	238 (84/154)	0.476
Age (year)	70.69 ± 8.86	67.11 ± 9.49	71.55 ± 8.50	<0.001
BMI (kg/m^2^)	25.29 ± 2.90	24.57 ± 2.44	25.46 ± 2.98	0.037
Waist circumference (cm)	90.79 ± 7.92	89.49 ± 6.94	91.1 ± 8.13	0.170
Hip circumference (cm)	101.01 ± 7.39	99.38 ± 6.34	101.41 ± 7.58	0.063
Waist-to-hip ratio (WHR)	0.90 ± 0.06	0.90 ± 0.05	0.90 ± 0.06	0.795
SBP (mmHg)	138.11 ± 14.66	136.53 ± 14.40	138.49 ± 14.73	0.365
DBP (mmHg)	78.41 ± 9.47	79.72 ± 9.62	78.1 ± 9.43	0.247
Obesity (BMI ≥ 25)	51.8% (153/142)	45.6% (26/31)	53.4% (127/111)	0.293
Fatty liver disease	74.9% (221/74)	66.7% (38/19)	76.9% (183/55)	0.045
Hypertension	91.5% (270/25)	87.7% (50/7)	92.4% (220/18)	0.251
Hyperlipidemia	41.0% (121/182)	42.1% (24/33)	39.9% (95/143)	0.762
Coronary heart disease	23.3% (69/226)	29.8% (17/40)	21.8% (52/186)	0.201
Cerebrovascular accident	0.07% (20/275)	0.07% (4/53)	0.07% (16/222)	0.937
Hyperuricemia	0.07% (22/273)	0.07% (4/53)	0.08% (18/220)	0.888
FPG (mmol/L)	7.60 ± 2.12	7.84 ± 2.36	7.55 ± 2.06	0.355
2 h PG (mmol/L)	11.37 ± 3.69	11.51 ± 3.52	11.33 ± 3.74	0.742
TG (mmol/L)	1.55 ± 0.93	1.51 ± 0.84	1.57 ± 0.96	0.675
HDL cholesterol (mmol/L)	1.33 ± 0.36	1.29 ± 0.25	1.34 ± 0.38	0.322
ALT (U/L)	25.26 ± 13.19	26.97 ± 13.84	24.85 ± 13.02	0.276
VLDL cholesterol (mmol/L)	2.57 ± 0.56	2.58 ± 0.59	2.57 ± 0.55	0.935

^a^ *P* value refers to the comparison between excess versus deficiency syndromes within the DM group using independent samples *t*-test for continuous variables and Pearson chi-square tests for categorical variable with the SPSS 17.0 software (SPSS, Chicago, Illinois, USA). *P* values < 0.05 were considered significant.

**Table 2:** Parameters from GA.

GA parameters	Initiate population	*K* ^a^	Selective ratio of initiate variable	Probability of simple point crossover

Excess versus deficiency	30	150	0.1	0.7

^a^ *K* means times of genetic algebra.

**Table 3:** Parameters from KOPLS models.

KOPLS Parameters	Sigma	Ao	ACCV^a^	R2X^b^	R2Y^b^	Q2Y^c^	Total accuracy	Balance accuracy	AUC^d^	AUC 95% confidence interval	sensitivity	specificity
Excess versus Deficency	2.5	3	0.860	0.425	1	0.944	0.949	0.968	0.968	0.950–0.987	1	0.937

^a^Accuracy of classification of cross-validation (ACCV) produced from each combination of *σ* and Ao parameters after cross-validation. ^b^R2Xcum and R2Ycum represent the cumulative sum of squares (SS) of all the X's and Y's explained by all extracted components. ^c^Q2Ycum is an estimate of how well the model predicts the Y's. ^d^AUC in 0.5~0.7 has lower accuracy, AUC in 0.7~0.9 has certain accuracy (model can be accepted), and AUC in more than 0.9 has high accuracy. When AUC = 0.5, the model has no value.
