# Supplementary material for: A Metabolomics Approach to Stratify Patients Diagnosed with Diabetes Mellitus into Excess or Deficiency Syndromes
Source: Evid Based Complement Alternat Med. 2015 Jan 18;2015:350703. doi: 10.1155/2015/350703 (PMC4312632; doi:10.1155/2015/350703)
Supplement: Supplementary file 1 — Figure S1 presents an overview of data analysis in the manuscript. Table S1 shows there is no significant difference of the four differential metabolites between groups with Mann-Whitney Test Analysis (P > 0.05). Furthermore, the KOPLS model also shows that no matter the age > 70 or ≤ 70 (n = 161 versus 134), deficiency group and excess group could be distinctly separated on the classification in Figure S2. Similar results are also found in BMI ≥ 25 or < 25 (n = 153 versus 142) in Figure S3.). The results prompts that the age and BMI with significant difference between deficiency and excess groups do not affect the final metabolomics results. [file 350703.f1.docx]

**Supplementary Materials**

Figure S1 An overview of data analysis.

Figure S2 K-OPLS model in DM patients with excess and deficiency syndrome (A) Age > 70 (n = 161), (B) Age ≤ 70 (n = 134).

Figure S3 K-OPLS model in DM patients with excess and deficiency syndrome (A) BMI ≥ 25 (n = 153), (B) BMI <25 (n = 142).

Table S1 Comparison of four differential metabolites in groups based on Mann-Whitney Test.


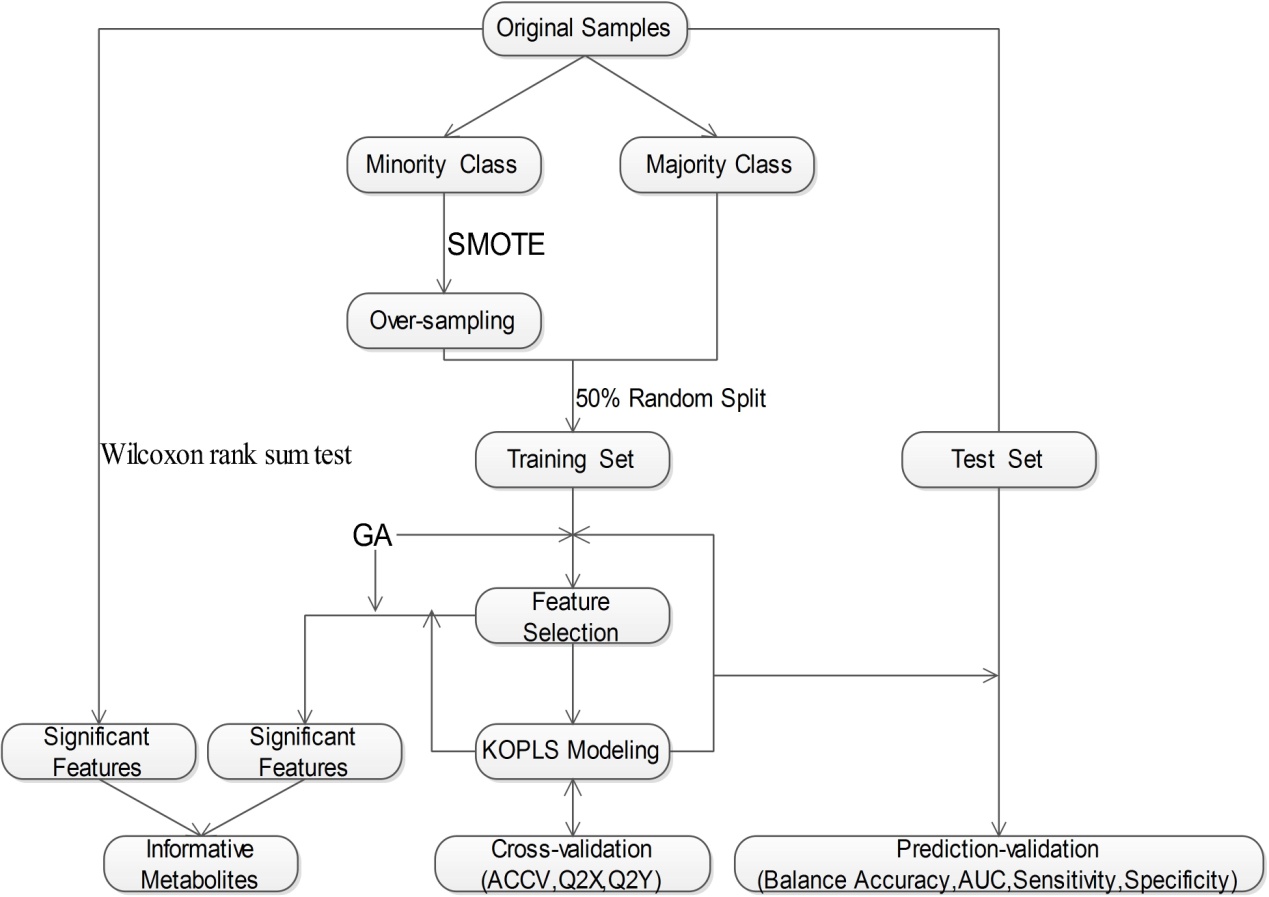


Figure S1 An overview of data analysis.


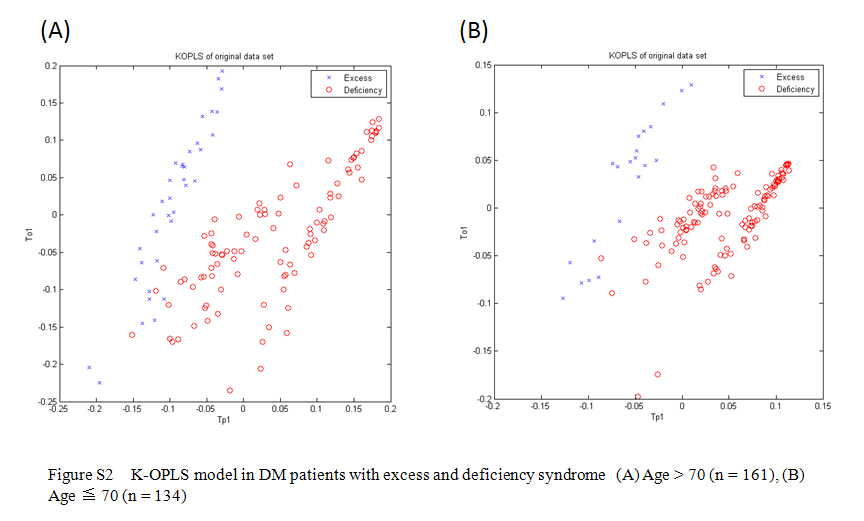


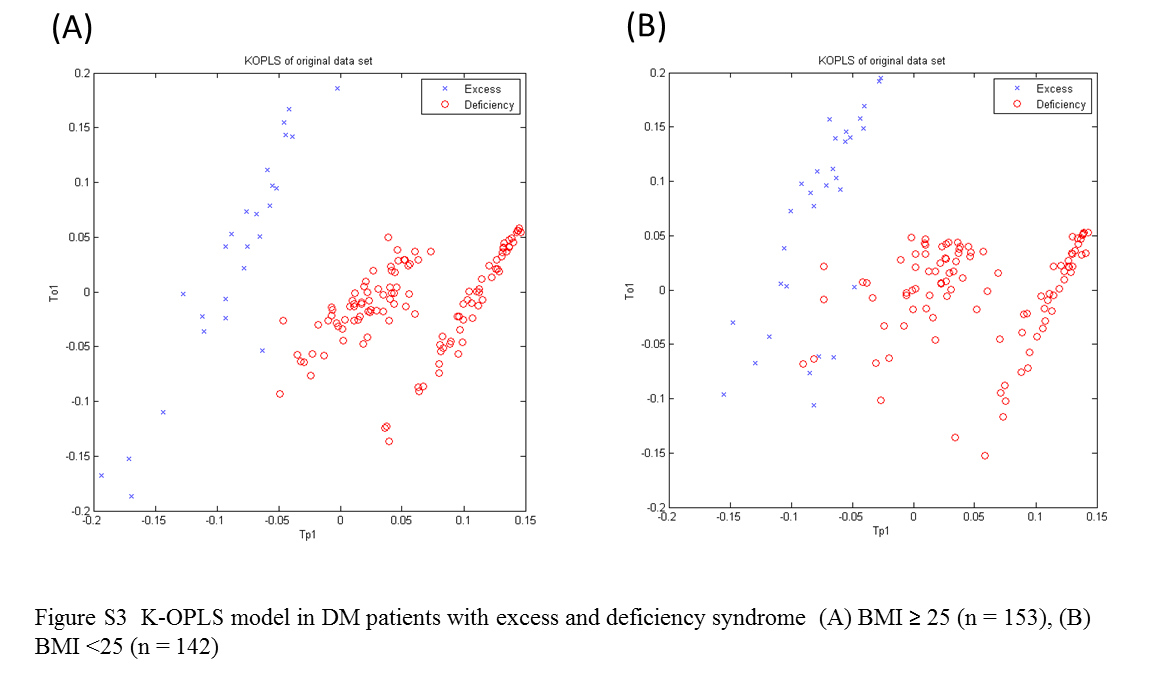


Table S1 Comparison of four differential metabolites in groups based on Mann-Whitney Test

| Metabolites* | Age(> 70,n=161) | Age(≤ 70,n=134) | p-value | Age(≥ 25,n=153) | BMI(< 25,n=142) | p-value |
| --- | --- | --- | --- | --- | --- | --- |
| 2-Indolecarboxylic acid | 4.47±0.85 | 3.71±0.47 | 0.462 | 3.87±0.55 | 4.40±0.88 | 0.602 |
| Hypotaurine | 31.20±3.83 | 39.83±5.17 | 0.173 | 39.64±5.30 | 30.25±3.17 | 0.136 |
| Pipecolinic acid | 36.40±2.00 | 38.65±2.30 | 0.459 | 39.64±2.28 | 35.05±1.94 | 0.129 |
| Progesterone | 9.80±1.04 | 9.42±1.16 | 0.806 | 9.75±1.03 | 9.49±1.16 | 0.868 |

* There is no significant difference among groups.
